# Elevated uric acid to serum albumin ratio: a predictor of short-term outcomes in Chinese heart failure patients

**DOI:** 10.3389/fnut.2024.1481155

**Published:** 2024-11-26

**Authors:** Xianling Liu, Aihui Chu, Xiahao Ding

**Affiliations:** ^1^Department of Cardiology, The First Affiliated Hospital of Nanjing Medical University, Nanjing, China; ^2^Department of Nursing, The First Affiliated Hospital of Nanjing Medical University, Nanjing, China; ^3^Department of Anesthesiology Nanjing Drum Tower Hospital, Medical School of Nanjing, Nanjing, China

**Keywords:** uric acid to serum albumin ratio (UAR), heart failure (HF), short-term outcomes, uric acid (UA), serum albumin (SA)

## Abstract

**Background:**

The prognostic value of the uric acid to albumin ratio (UAR) in heart failure (HF) remains underexplored. The objective of this research was to investigate the link between UAR and short-term outcomes in Chinese HF patients.

**Methods:**

We analyzed data from 1893 HF patients, out of an initial cohort of 2008, who had available UAR measurements. The skewed distribution of UAR data was addressed by applying a Log-10 (lg) transformation and stratifying patients into three groups accordingly (low to high). The final outcome was identified as mortality or hospital readmission within 28 days. We employed restricted cubic spline analysis (RCS), Kaplan–Meier survival curves, and Cox proportional hazards models to evaluate the link between UAR and short-term outcomes.

**Results:**

Among 1893 patients with HF [≥ 70 years, 1,382 (73.0%); female, 1,100 (58.1%)], the incidence of 28-day outcome was 8.6%. The RCS analysis suggested a positive relationship between lg(UAR) and 28-day outcomes, with no evidence of nonlinearity (*p* = 0.008). The cumulative incidence of 28-day readmission/death indicated that patients in the tertile 3 faced a significantly elevated risk of adverse outcomes (*p* < 0.001). Cox proportional hazards models showed that an elevated UAR was associated with a greater likelihood of 28-day mortality or hospital readmission (HR = 2.433, 95% CI: 1.638–3.615, *p* < 0.001). Even after accounting for possible confounding variables, the result still existed (HR = 1.594, 95% CI: 1.032–2.462, *p* = 0.036). Moreover, the associations were consistent in various subgroups, and sensitivity analysis (all *p* > 0.05).

**Conclusion:**

Increased UAR correlates with a heightened risk of short-term death or hospital readmission in Chinese individuals suffering from HF. Maintaining a relatively lower UAR could potentially improve the clinical prognosis for these patients.

## 1 Introduction

Heart failure (HF) is the terminal manifestation of cardiovascular disease, characterized by a reduction in cardiac output caused by abnormal ventricular filling and ejection, which cannot meet metabolic demands. It is a common clinical and public health issue (1). As the population ages and chronic conditions like hypertension and coronary artery disease (CAD) become more prevalent, hospitalizations and deaths due to HF have increased (2). Despite recent advancements in the management and treatment of HF, readmission rates and mortality remain persistently high. Moreover, the costs associated with readmissions account for a significant portion of total HF-related expenditures, severely impacting the quality of life and placing a substantial economic burden on healthcare systems (3). In order to maximize treatment, improve symptoms, and increase survival, it is imperative to identify HF patients at risk for poor outcomes as early as possible.

New biomarkers are being evaluated for their ability to advance the management of patients with heart failure. Despite a large pool of promising candidate biomarkers, the complex pathophysiology of HF has limited their acceptance and application in clinical settings (4). Several biomarkers, including B-type natriuretic peptide (BNP), N-terminal pro-B-type natriuretic peptide (NT-proBNP), high-sensitivity cardiac troponin (hs-cTn), and C-reactive protein (CRP), have been shown to predict HF prognosis (5). However, each has limitations. BNP and NT-proBNP tests are relatively costly and are influenced by factors such as age, sex, body weight, and renal function, which can reduce their specificity and stability (6). Additionally, hs-cTn and CRP levels can be elevated in conditions such as cerebrovascular disease, sepsis, chronic kidney disease, connective tissue disorders, and malignancies, resulting in low specificity (5).

Studies have shown that elevated serum uric acid (UA) levels and hyperuricemia are implicated in the pathogenesis of several diseases, including cardiovascular disease (CVD) and chronic kidney disease (CKD) (7–9). In HF patients, elevated UA levels have been linked to poor prognosis, with higher levels significantly correlated with increased all-cause mortality (10, 11). Besides, hypoalbuminemia has also been found to be prevalent in HF patients, with its incidence positively correlated with age and disease severity (12). In addition to predicting HF occurrence, serum albumin (SA) levels also act as an independent prognostic factor, allowing risk stratification of HF patients and identifying those at high cardiovascular risk (13). The uric acid-to-albumin ratio (UAR) outperforms single UA or SA levels in predicting the severity and extent of acute coronary syndrome and demonstrates higher sensitivity and specificity for predicting adverse outcomes (14–16). With HF being a heterogeneous clinical syndrome, a multi-biomarker and multimodality approach is recognized as an appealing strategy to incorporate different pathological pathways and improve risk prediction (5).

In light of the importance of elevated UA levels, decreased albumin levels, and cardiovascular disease, we hypothesize that the UAR, calculated as the ratio of uric acid to albumin, could serve as a biomarker that comprehensively reflects oxidative stress, inflammatory response, and nutritional status, thereby predicting short-term prognosis in HF patients. Several studies have already shown significant associations between this composite marker and mortality and disease severity, including CAD (myocardial infarction) (15, 17), aortic dissection (18), stroke (19), and acute kidney injury (20). It is unclear, however, whether UAR is associated with the short-term prognosis of patients with heart failure. We aimed to investigate the predictive significance of UAR for short-term outcomes in HF patients. Subsequently, medical providers would be able to use UAR as a more accurate tool in better managing HF patients by examining the link between UAR and short-term outcome.

## 2 Methods

### 2.1 Study design and data source

This study utilized data from an open-access, single-center, retrospective database available on the PhysioNet platform (21). The database contains records from December 2016 to June 2019 and includes consultation and follow-up information for 2008 HF patients admitted to Zigong Fourth People’s Hospital in Sichuan, China (22). All patients in the database are of Chinese ethnicity, which provides a homogenous sample for analysis. This retrospective cohort study analyzed over 150 variables, encompassing patient demographics, comorbidities, laboratory and imaging results, outcome events, and medical records. According to the European Society of Cardiology (ESC) (23), HF is diagnosed by using the criteria established by the ESC. The study adhered to the STROBE (Strengthening the Reporting of Observational Studies in Epidemiology) guidelines and received approval from the Ethics Committee of Zigong Fourth People’s Hospital (approval number: 2020–010). Informed consent was waived due to the retrospective nature of the study, which followed the Helsinki Declaration guidelines.

### 2.2 Independent variable and outcome

In this study, UAR was measured as mg/g, calculated by dividing uric acid levels (mg/dL) by serum albumin levels (g/dL). To address the skewed distribution of UAR data, a Log-10 (lg) transformation was applied (24), and patients were stratified into three groups based on tertiles of lg(UAR): tertile 1: lg(UAR) < 0.251, tertile 2: lg(UAR) 0.251–0.398, and tertile 3: lg(UAR) ≥ 0.398.

The measured outcomes comprised the 28-day mortality, the 28-day hospital readmission rate, the duration from admission to death (in days), and the interval from initial admission to subsequent readmission (in days). Study outcomes included mortality and readmission within 28 days, both defined as a composite outcome.

### 2.3 Study population

The study population consisted of adult patients diagnosed with HF, selected from the database. Exclusion criteria were as follows: (1) missing data on UA or SA levels, and (2) lack of records regarding readmission time or death time.

### 2.4 Covariates

Upon admission, comprehensive clinical characteristics and medical histories were collected for each patient. These included age, sex, body mass index (BMI), New York Heart Association (NYHA) cardiac function classification, systolic blood pressure (SBP), diastolic blood pressure (DBP), and histories of myocardial infarction (MI), cerebrovascular disease (CVD), diabetes mellitus (DM), chronic kidney disease (CKD), chronic obstructive pulmonary disease (COPD), and liver disease (LD). Laboratory results were obtained within the first 24 h of admission, encompassing white blood cell count (WBC), neutrophil count, monocyte count, lymphocyte count, red blood cell count (RBC), hemoglobin (Hb), platelet count, potassium, sodium, total cholesterol (TC), low-density lipoprotein cholesterol (LDL-C), high-density lipoprotein cholesterol (HDL-C), triglycerides (TG), albumin, urea, creatinine, uric acid (UA), estimated glomerular filtration rate (eGFR), high-sensitivity cardiac troponin (hs-cTn), and brain natriuretic peptide (BNP). Additionally, medications administered during hospitalization, including angiotensin-converting enzyme inhibitors/angiotensin receptor blockers (ACEI/ARB), *β*-receptor blockers, diuretics, inotropes, vasodilators, statins, anticoagulants, and antiplatelet agents, were meticulously documented.

### 2.5 Statistical analysis

All statistical analyses were conducted using R software (version 4.4.1, R Foundation for Statistical Computing, Vienna, Austria), MSTATA software,^1^ and IBM SPSS Statistics for Windows (version 21.0, SPSS Inc., Chicago, Illinois, United States). Statistical significance was defined as a two-sided *P* - value of less than 0.05.

Descriptive statistics for continuous variables were reported as mean ± standard deviation (SD) or median with interquartile range (IQR), depending on the distribution. Categorical variables were summarized as frequencies or proportions. To compare baseline characteristics among the three UAR groups, we employed one-way analysis of variance (ANOVA) for continuous variables and the Chi-square test or Fisher’s exact test, as appropriate, for categorical variables.

The relationship between lg(UAR) and the risk of all-cause mortality or readmission within 28 days was assessed using multivariable-adjusted restricted cubic spline (RCS) regression, with three knots placed at the 10th, 50th, and 90th percentiles of lg(UAR). Kaplan–Meier survival analysis was used to estimate the incidence of all-cause mortality or readmission within 28 days, with comparisons made using the log-rank test. To further explore the association between lg(UAR) and 28-day poor prognosis risk, we conducted multivariable Cox proportional hazards regression analyses. Four adjusted models were developed: Model 2 included adjustments for age, sex, and BMI; Model 3 further adjusted for specific comorbidities; Model 4 expanded upon Model 3 by including medication status; and Model 5 incorporated all variables from Model 4, with additional adjustments for laboratory variables such as hs-cTn and BNP. Our findings were validated via stratification of baseline lg(UAR) into three categories (low to high) and by calculating the trend *p*-value, we assessed the robustness of HR estimates.

To determine whether the association between lg(UAR) and 28-day readmission or mortality risk was consistent across different subpopulations, we conducted interaction and subgroup analyses based on variables such as age, sex, BMI, NYHA cardiac function classification, MI, CVD, DM, CKD, and history of ACEI/ARB and *β*-receptor blocker use. These results were visualized using a forest plot, and the likelihood ratio test was used to assess interactions between subgroups. Given the significant role of left ventricular ejection fraction (LVEF) in predicting HF outcomes and the high prevalence of missing LVEF data, we also performed sensitivity analyses to account for these missing data.

### 2.6 Handling of missing data

In our study, the random forest method, known for its flexibility and robust predictive capability, was employed to address the issue of missing data. Given the presence of multiple missing variables, including BNP (1.2%) and LVEF (68.1%), this method was chosen for imputation, as it is widely recognized for effectively managing complex datasets with high proportions of missing data (25).

## 3 Results

### 3.1 Baseline characteristics

**Figure 1:**
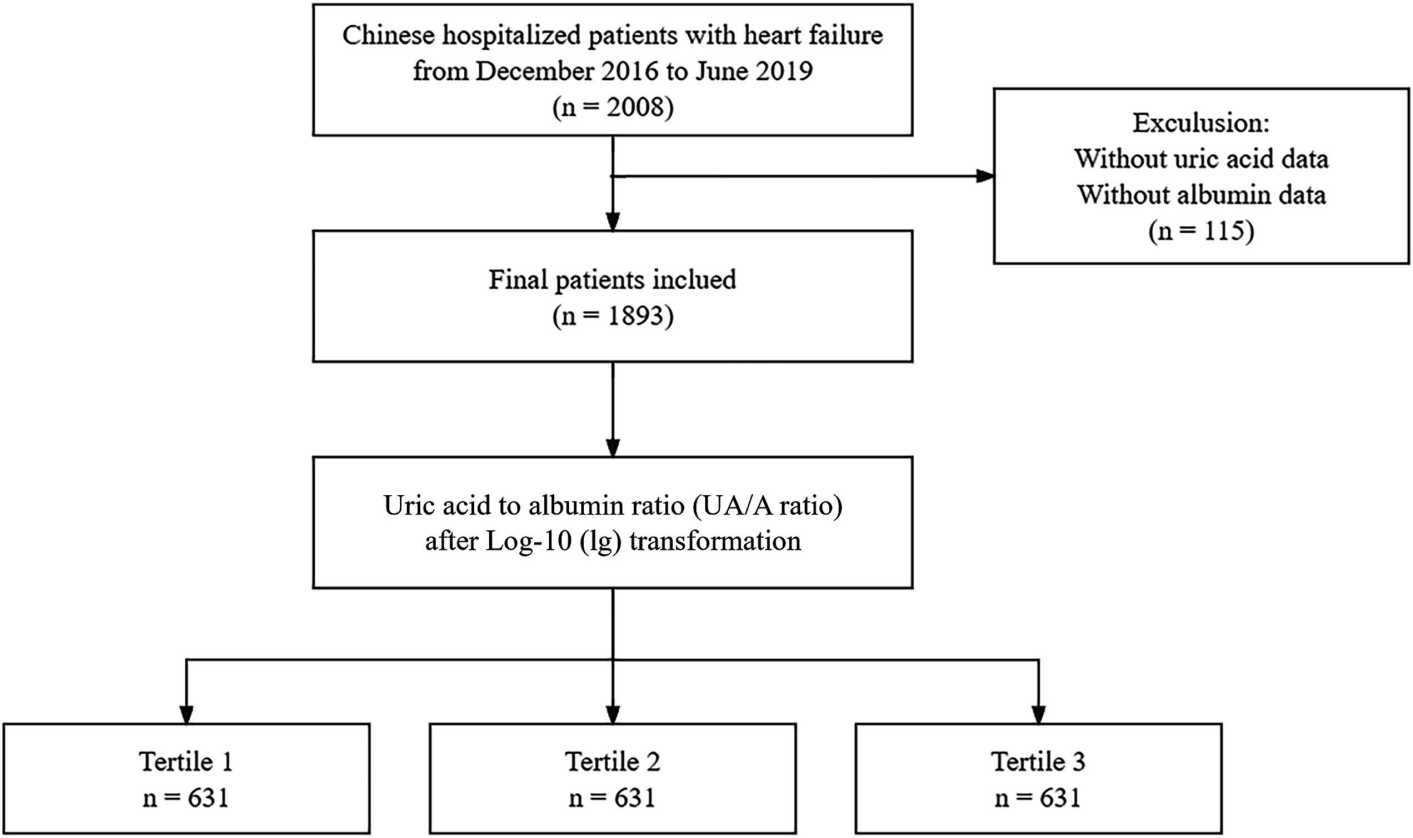
Flow chart of the study population and exclusion criteria.

After excluding 115 individuals due to missing SA and UA values, the final analysis dataset consisted of 1893 patients, as shown in Figure 1. Table 1 provides a summary of the demographic and clinical characteristics of these patients, stratified by tertiles of lg(UAR). A significant proportion of the study population were over the age of 70 (73%, *n* = 1,382) and female (58.1%, *n* = 1,100). Compared with patients in the lower tertiles of lg(UAR; Tertile 1 and Tertile 2), those in the highest tertile (Tertile 3) were more likely to be female and exhibited a higher NYHA classification, indicating more advanced cardiac dysfunction. They also demonstrated significantly lower SBP and DBP (all *p* < 0.05). Additionally, Tertile 3 patients had a higher prevalence of CKD and LD, and were more frequently administered diuretics and inotropes during hospitalization, with lower rates of ACEI/ARB use (all *p* < 0.05). Biochemically, patients in the highest lg(UAR) tertile showed elevated levels of WBC, hs-cTn, BNP, urea, creatinine, UA, and serum potassium. Conversely, these patients had significantly reduced levels of RBC, SA, eGFR, serum sodium, and lipid profile markers (e.g., HDL, LDL; all *p* < 0.05), as summarized in Table 2.

**Table 1:** Baseline demographic data of patients with heart failure stratified by tertiles of lg(UAR).

Characteristics	Overall *N* = 1,893	Tertiles of lg(UAR), mg/g			
		Tertile 1 *N* = 631	Tertile 2 *N* = 631	Tertile 3 *N* = 631	*p*-value
Outcome	162 (8.6%)	35 (5.5%)	45 (7.1%)	82 (13.0%)	<0.001
Age, years					0.541
<70	511 (27.0%)	168 (26.6%)	163 (25.8%)	180 (28.5%)	
≥70	1,382 (73.0%)	463 (73.4%)	468 (74.2%)	451 (71.5%)	
Sex, n (%)					<0.001
Female	1,100 (58.1%)	448 (71.0%)	354 (56.1%)	298 (47.2%)	
Male	793 (41.9%)	183 (29.0%)	277 (43.9%)	333 (52.8%)	
BMI, kg/m^2^					0.254
<18.5	478 (25.3%)	157 (24.9%)	148 (23.5%)	173 (27.4%)	
[18.5, 23.9]	1,120 (59.2%)	364 (57.7%)	391 (62.0%)	365 (57.8%)	
[24, 27.9]	230 (12.2%)	91 (14.4%)	69 (10.9%)	70 (11.1%)	
≥28	65 (3.4%)	19 (3.0%)	23 (3.6%)	23 (3.6%)	
NYHA, n (%)					<0.001
II	331 (17.5%)	125 (19.8%)	113 (17.9%)	93 (14.7%)	
III	983 (51.9%)	350 (55.5%)	333 (52.8%)	300 (47.5%)	
IV	579 (30.6%)	156 (24.7%)	185 (29.3%)	238 (37.7%)	
SBP, mmHg	131 ± 25	137 ± 25	133 ± 24	124 ± 23	<0.001
DBP, mmHg	77 ± 14	80 ± 14	77 ± 14	73 ± 14	<0.001
MI, n (%)	136 (7.2%)	40 (6.3%)	38 (6.0%)	58 (9.2%)	0.056
CVD, n (%)	142 (7.5%)	43 (6.8%)	56 (8.9%)	43 (6.8%)	0.276
DM, n (%)	439 (23.2%)	133 (21.1%)	151 (23.9%)	155 (24.6%)	0.295
CKD, n (%)	447 (23.6%)	65 (10.3%)	139 (22.0%)	243 (38.5%)	<0.001
LD, n (%)	81 (4.3%)	22 (3.5%)	20 (3.2%)	39 (6.2%)	0.015
COPD, n (%)	224 (11.8%)	76 (12.0%)	79 (12.5%)	69 (10.9%)	0.670
Diuretics, n (%)	1,862 (98.4%)	613 (97.1%)	620 (98.3%)	629 (99.7%)	0.002
Inotropes, n (%)	1,663 (87.8%)	533 (84.5%)	548 (86.8%)	582 (92.2%)	<0.001
Vasodilators, n (%)	1,108 (58.5%)	358 (56.7%)	385 (61.0%)	365 (57.8%)	0.278
ACEI/ARB, n (%)	730 (38.6%)	270 (42.8%)	236 (37.4%)	224 (35.5%)	0.022
Β-blockers, n (%)	708 (37.4%)	243 (38.5%)	242 (38.4%)	223 (35.3%)	0.423
Statins, n (%)	784 (41.4%)	269 (42.6%)	278 (44.1%)	237 (37.6%)	0.048
Antiplatelet agents, n (%)	1,137 (60.1%)	379 (60.1%)	397 (62.9%)	361 (57.2%)	0.118
Anticoagulants, n (%)	451 (23.8%)	155 (24.6%)	162 (25.7%)	134 (21.2%)	0.157

BMI, body mass index; NYHA, New York Heart Association; SBP, systolic blood pressure; DBP, diastolic blood pressure; MI, myocardial infarction; CVD, cerebrovascular disease; DM, diabetes mellitus; CKD, chronic kidney disease; COPD, chronic obstructive pulmonary disease; LD, liver disease; ACEI/ARB, angiotensin-converting enzyme inhibitor/angiotensin receptor blocker; Β-blocker, β-receptor blockers. *p-*values were obtained through one-way ANOVA analysis for continuous variables and chi-square test for categorical variables.

**Table 2:** Baseline laboratory characteristics of patients with heart failure stratified by tertiles of lg(UAR).

Characteristics	Overall *N* = 1,893	Tertiles of lg(UAR), mg/g			
		Tertile 1 *N* = 631	Tertile 2 *N* = 631	Tertile 3 *N* = 631	*p*-value
WBC, *10^9/L	6.5 (5.1, 8.7)	6.2 (4.9, 8.1)	6.5 (5.2, 8.3)	7.0 (5.1, 9.4)	<0.001
Neutrophil, *10^9/L	4.84 (3.62, 6.80)	4.55 (3.45, 6.25)	4.87 (3.74, 6.40)	5.24 (3.67, 7.36)	<0.001
Monocyte, *10^9/L	0.42 (0.32, 0.57)	0.39 (0.30, 0.51)	0.43 (0.32, 0.56)	0.46 (0.33, 0.64)	<0.001
Lymphocyte, *10^9/L	0.94 (0.62, 1.29)	0.98 (0.67, 1.32)	0.94 (0.62, 1.27)	0.90 (0.57, 1.28)	0.014
RBC, *10^12/L	3.85 ± 0.76	3.94 ± 0.63	3.86 ± 0.76	3.75 ± 0.86	<0.001
Hb, g/L	115 ± 24	118 ± 21	115 ± 24	111 ± 27	<0.001
Platelet, *10^9/L	135 (101, 176)	135 (102, 177)	135 (102, 172)	133 (97, 177)	0.654
hs-cTn, pg./mL	0.06 (0.02, 0.12)	0.03 (0.01, 0.08)	0.05 (0.02, 0.11)	0.09 (0.05, 0.19)	<0.001
BNP, pg./mL	781 (327, 1,784)	461 (186, 1,006)	801 (341, 1,707)	1,246 (627, 2,624)	<0.001
Albumin, g/L	36.5 ± 5.0	38.6 ± 4.4	36.7 ± 4.3	34.3 ± 5.2	<0.001
TC, mmol/L	3.61 (3.01, 4.28)	3.84 (3.30, 4.58)	3.71 (3.10, 4.31)	3.28 (2.64, 3.95)	<0.001
LDL, mmol/L	1.76 (1.36, 2.27)	1.85 (1.43, 2.37)	1.80 (1.41, 2.28)	1.64 (1.22, 2.11)	<0.001
TG, mmol/L	0.97 (0.73, 1.30)	0.97 (0.72, 1.31)	0.98 (0.74, 1.30)	0.97 (0.73, 1.29)	0.978
HDL, mmol/L	1.08 (0.87, 1.29)	1.20 (1.00, 1.40)	1.10 (0.93, 1.30)	0.91 (0.74, 1.12)	<0.001
Creatinine, umol/L	87 (65, 124)	65 (54, 82)	89 (69, 113)	123 (91, 166)	<0.001
Urea, mmol/L	8.1 (5.9, 11.6)	6.1 (4.8, 7.8)	8.0 (6.0, 10.7)	11.6 (8.5, 15.9)	<0.001
UA, umol/L	458 (363, 572)	335 (285, 374)	462 (414, 510)	637 (558, 743)	<0.001
eGFR, mL/min/1.73 m^2^	65 (41, 90)	88 (67, 108)	64 (45, 83)	43 (30, 63)	<0.001
lg(UAR), mg/g	0.33 ± 0.17	0.15 ± 0.09	0.32 ± 0.04	0.51 ± 0.09	<0.001
Potassium, mmol/L	3.87 (3.52, 4.32)	3.73 (3.44, 4.04)	3.91 (3.56, 4.38)	4.06 (3.58, 4.56)	<0.001
Sodium, mmol/L	139.0 (136.0, 141.4)	139.9 (137.1, 142.0)	139.0 (136.5, 141.6)	137.8 (134.8, 140.4)	<0.001

WBC, white blood cell count; RBC, red blood cell count; Hb, hemoglobin; hs-cTn, high-sensitivity cardiac troponin; BNP, brain natriuretic peptide; TC, total cholesterol; LDL, low-density lipoprotein cholesterol; HDL, high-density lipoprotein cholesterol; TG, triglycerides; UA, uric acid; eGFR, estimated glomerular filtration rate; UAR, uric acid to albumin ratio. *p-*values were obtained through one-way ANOVA analysis for continuous variables and chi-square test for categorical variables.

### 3.2 Association between lg(UAR) and clinical outcomes

**Figure 2:**
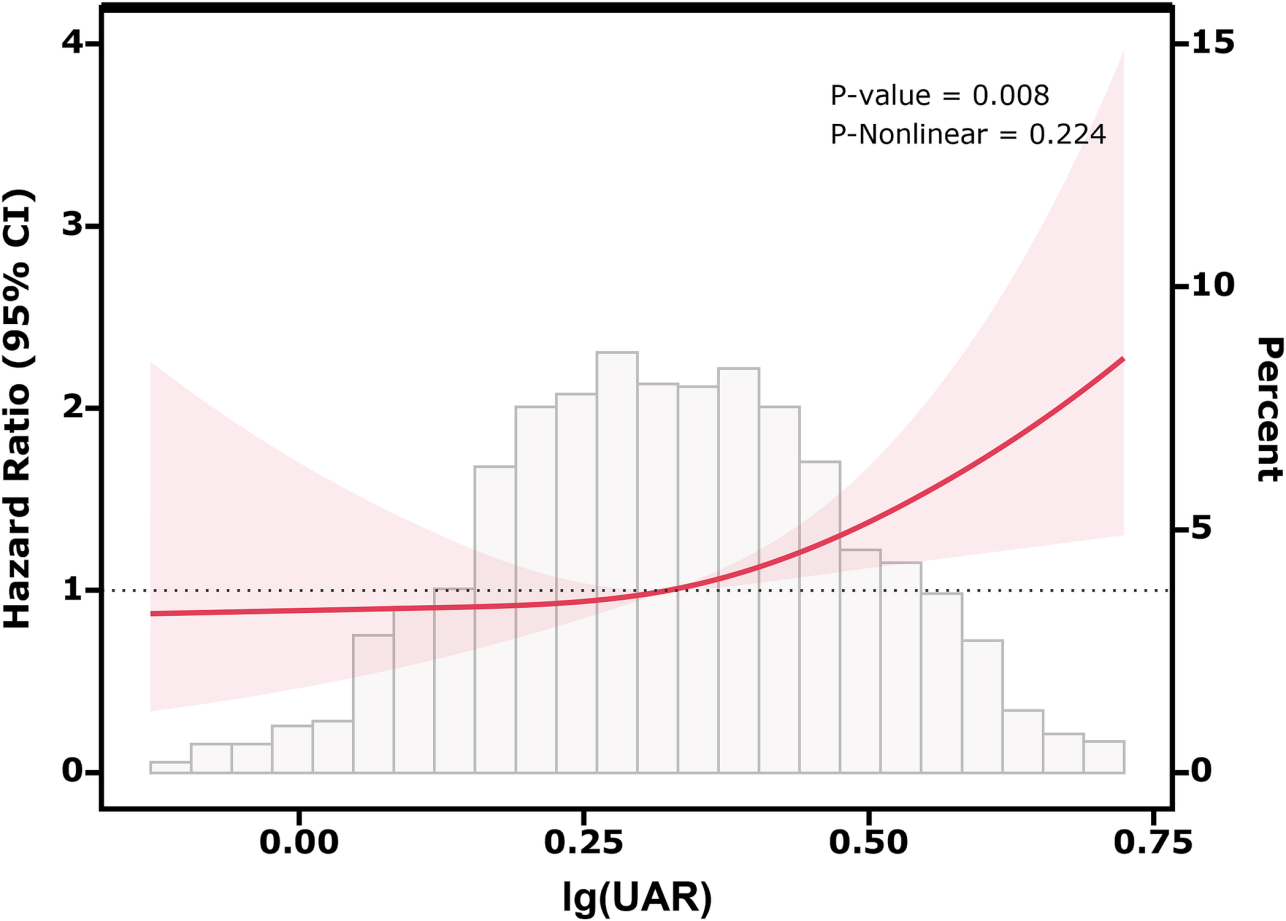
Relationship between lg(UAR) and the risk of 28-day composite outcomes, including all-cause mortality and readmission, based on restricted cubic spline curves. Adjusted for diuretics, inotropes, vasodilators, ACEI/ARB, β-receptor blockers, statins, anticoagulants, antiplatelet agents, age, sex, BMI, NYHA, SBP, DBP, MI, CVD, DM, CKD, LD, COPD, hs-cTn, BNP. Abbreviations: UAR, uric acid to albumin ratio; ACEI/ARB, angiotensin-converting enzyme inhibitor/angiotensin receptor blocker; BMI, body mass index; NYHA, New York Heart Association; SBP, systolic blood pressure; DBP, diastolic blood pressure; MI, myocardial infarction; CVD, cerebrovascular disease; DM, diabetes mellitus; CKD, chronic kidney disease; COPD, chronic obstructive pulmonary disease; LD, liver disease; hs-cTn, high-sensitivity cardiac troponin; BNP, brain natriuretic peptide.

Figure 2 shows the results of the RCS analysis, illustrating the association between lg(UAR) and 28-day adverse prognosis in HF patients. The RCS model was adjusted for multiple confounders, including use of diuretics, inotropes, vasodilators, ACEI/ARB, *β*-receptor blockers, statins, anticoagulants, and antiplatelet agents, as well as patient-specific factors such as age, sex, BMI, NYHA, SBP, DBP, hs-cTn, BNP, and comorbidities (MI, CVD, DM, CKD, LD, COPD). The analysis revealed a statistically significant positive association between lg(UAR) and the likelihood of adverse outcomes within 28 days (*P* for overall association = 0.008), with no significant nonlinearity observed (*P* for nonlinearity = 0.224). A notable threshold effect was observed, with the risk of adverse outcomes increasing significantly when lg(UAR) reached or exceeded 0.324 (corresponding to UAR = 2.11 mg/g). At this threshold, the hazard ratio for adverse events was 1.40 (95% CI: 1.21–1.62, *p* < 0.001), indicating a 40% increase in risk. These results suggest that higher lg(UAR) levels are associated with a progressively increased risk of short-term adverse outcomes in HF patients (Supplementary Table 1).

**Figure 3:**
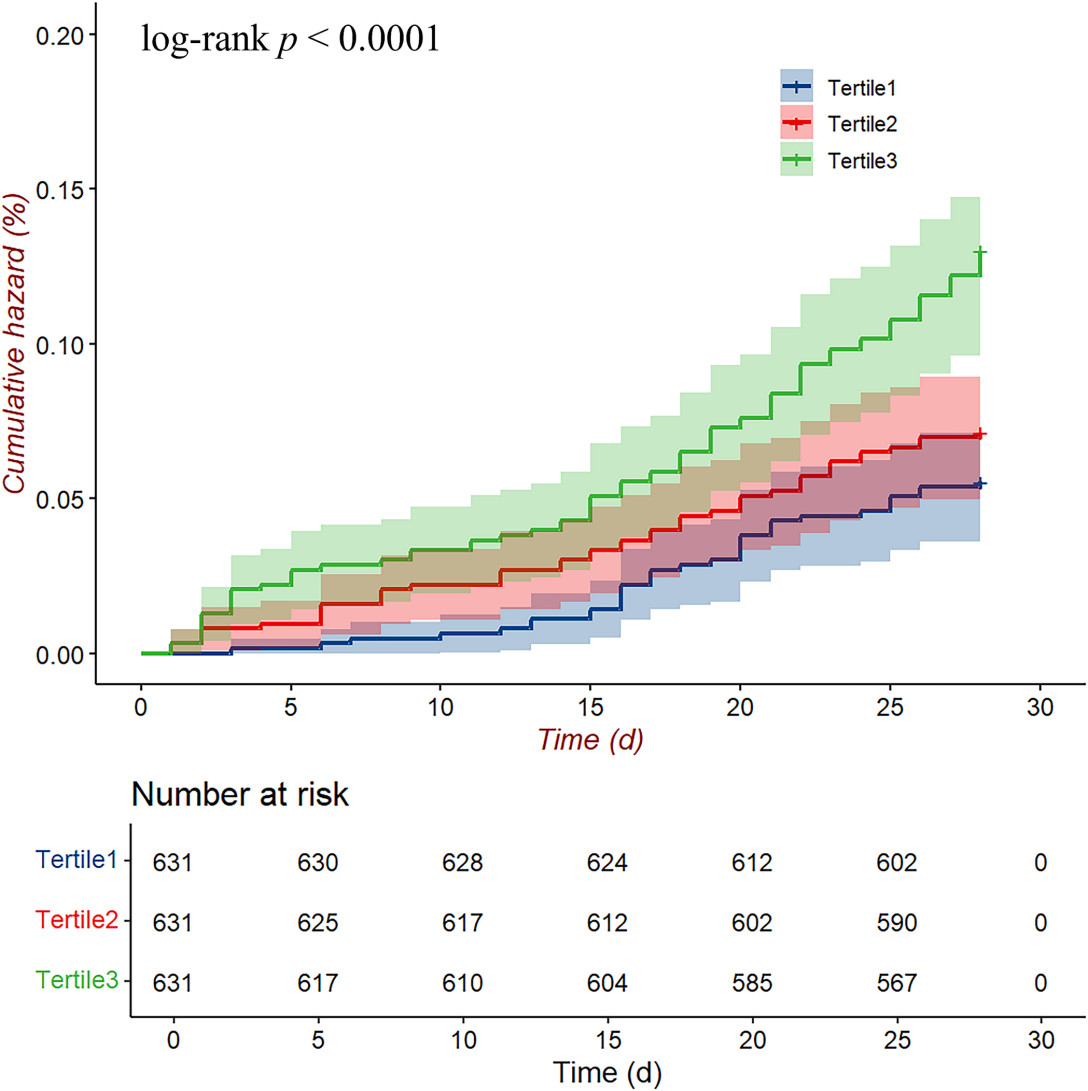
Cumulative hazard curves for the 28-day composite endpoint (all-cause mortality and readmission) according to lg(UAR) levels. Abbreviations: UAR, uric acid to albumin ratio.

Figure 3 presents the 28-day cumulative hazard of readmission or death among HF patients stratified by lg(UAR) tertiles. Patients in the highest tertile (Tertile 3, green line) showed a significantly higher cumulative hazard compared to those in the middle (Tertile 2, red line) and lowest tertiles (Tertile 1, blue line), as confirmed by the log-rank test (*p* < 0.001). By the end of the 28-day period, the cumulative hazard for Tertile 3 was clearly elevated relative to the other groups, with Tertile 1 consistently showing the lowest cumulative hazard.

Table 3 summarizes the association between lg(UAR) and clinical outcomes across different models. In the unadjusted model (Model 1), each one-unit increase in lg(UAR) was associated with a 51.3% higher risk of adverse outcomes (HR = 1.513, 95% CI: 1.299–1.762, *p* < 0.001). This association remained significant across all adjusted models, with HR values decreasing as additional confounders were controlled. In the fully adjusted model (Model 5), the HR was 1.255 (95% CI: 1.058–1.488, *p* = 0.009), indicating that lg(UAR) independently predicts adverse outcomes. When analyzed by tertiles, patients in Tertile 3 showed a significantly higher risk of adverse outcomes than those in Tertile 1. In Model 1, the risk in Tertile 3 was 2.433 times higher than in Tertile 1 (95% CI: 1.638–3.615, *p* < 0.001). This increased risk persisted after full adjustment in Model 5, with an HR of 1.594 (95% CI: 1.032–2.462, *p* = 0.036). Trend analysis (*P* for trend <0.05 in all models) further confirmed that higher lg(UAR) tertiles are associated with a progressively increased risk of adverse outcomes. Lg(UAR) is an independent predictor of short-term adverse outcomes in HF patients, both as a continuous variable and when categorized into tertiles, with the strongest association observed in the highest tertile.

**Table 3:** Association between lg(UAR) and clinical outcomes in patients with heart failure.

Variables	Model 1		Model 2		Model 3		Model 4		Model 5	
	HR (95%CI)	*p*	HR (95%CI)	*p*	HR (95%CI)	*p*	HR (95%CI)	*p*	HR (95%CI)	*p*
lg(UAR)	1.513 (1.299, 1.762)	<0.001	1.495 (1.279, 1.747)	<0.001	1.325 (1.120, 1.569)	0.001	1.280 (1.085, 1.509)	0.003	1.255 (1.058, 1.488)	0.009
lg(UAR) Tertiles										
Tertile1	1.000 (Reference)		1.000 (Reference)		1.000 (Reference)		1.000 (Reference)		1.000 (Reference)	
Tertile2	1.304 (0.838, 2.028)	0.239	1.269 (0.814, 1.978)	0.294	1.150 (0.735, 1.799)	0.541	1.130 (0.722, 1.768)	0.593	1.110 (0.708, 1.739)	0.648
Tertile3	2.433 (1.638, 3.615)	<0.001	2.332 (1.559, 3.489)	<0.001	1.768 (1.156, 2.705)	0.009	1.674 (1.095, 2.557)	0.017	1.594 (1.032, 2.462)	0.036
*P* for trend		<0.001		<0.001		0.005		0.009		0.020

Model 1: Crude. Model 2: Adjust: AGE, SEX, BMI. Model 3: Adjust: AGE, SEX, BMI, NYHA, SBP, DBP, MI, CVD, DM, CKD, LD, COPD. Model 4: Adjust: diuretic, inotropes, vasodilator, ACEI/ARB, Β - blocker, anticoagulant, antiplatelet, statin, AGE, SEX, BMI, NYHA, SBP, DBP, MI, CVD, DM, CKD, LD, COPD. Model 5: Adjust: diuretic, inotropes, vasodilator, ACEI/ARB, Β - blocker, anticoagulant, antiplatelet, statin, AGE, SEX, BMI, NYHA, SBP, DBP, MI, CVD, DM, CKD, LD, COPD, hs-cTn, BNP. HR, Hazard Ratio; CI, Confidence Interval; BMI, body mass index; NYHA, New York Heart Association; SBP, systolic blood pressure; DBP, diastolic blood pressure; MI, myocardial infarction; CVD, cerebrovascular disease; DM, diabetes mellitus; CKD, chronic kidney disease; COPD, chronic obstructive pulmonary disease; LD, liver disease; hs-cTn, high-sensitivity cardiac troponin; BNP, brain natriuretic peptide; UAR, uric acid to albumin ratio; ACEI/ARB, angiotensin-converting enzyme inhibitor/angiotensin receptor blocker; Β-blocker, β-receptor blockers.

### 3.3 Sensitivity analyses

**Figure 4:**
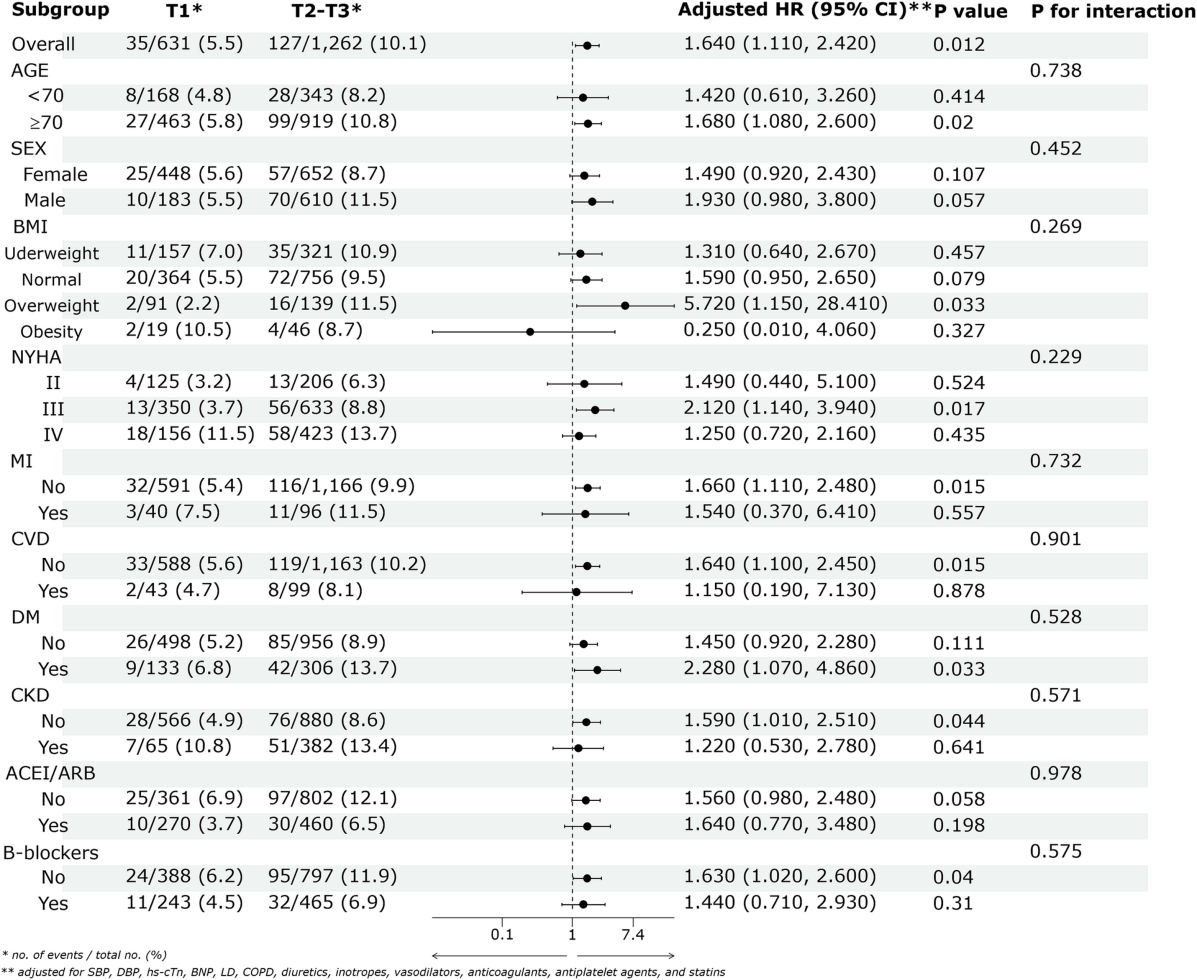
The association between lg(UAR) and the risk of the 28-day composite outcome of all-cause mortality and readmission across various subgroups. Adjusted for SBP, DBP, cTn, BNP, LD, COPD, diuretics, inotropes, vasodilators, anticoagulants, antiplatelet agents, and statins. Abbreviations: UAR, uric acid to albumin ratio; ACEI/ARB, angiotensin-converting enzyme inhibitor/angiotensin receptor blocker; BMI, body mass index; NYHA, New York Heart Association; SBP, systolic blood pressure; DBP, diastolic blood pressure; MI, myocardial infarction; CVD, cerebrovascular disease; DM, diabetes mellitus; CKD, chronic kidney disease; COPD, chronic obstructive pulmonary disease; LD, liver disease; hs-cTn, high-sensitivity cardiac troponin; BNP, brain natriuretic peptide; T1, Tertile 1; T2, Tertile 2; T3, Tertile 3.

Figure 4 displays the forest plot of adjusted HRs with 95% CIs for the association between lg(UAR) and 28-day adverse outcomes in HF patients across various subgroups. Patients in the higher lg(UAR) tertiles (Tertile 2 and Tertile 3) demonstrated a significantly increased risk of adverse outcomes compared to those in the lowest tertile (Tertile 1), with an adjusted HR of 1.640 (95% CI: 1.110–2.420, *p* = 0.012). No significant interaction effects were observed among the subgroups, indicating that the increased risk associated with higher lg(UAR) levels was consistent across different subgroup characteristics (all *p* > 0.05). Sensitivity analyses confirmed that the association between lg(UAR) and adverse clinical outcomes was not influenced by variations in LVEF (*P* for interaction = 0.856; Supplementary Figure 1; Supplementary Table 2).

## 4 Discussion

In this retrospective cohort study of 1893 patients with HF, we investigated the association between lg(UAR) and patient prognosis. Our findings indicate that lg(UAR) is a significant predictor of adverse outcomes in HF patients. Specifically, patients with higher lg(UAR) levels exhibited increased rates of mortality and rehospitalization, suggesting that lg(UAR) could serve as a valuable prognostic marker in clinical practice. The consistency observed in subgroup analyses further supports the robustness and reliability of these conclusions.

In the present study, we found that UAR is a significant independent predictor of short-term adverse outcomes in HF patients. HF is characterized by a complex pathological process in which patients often experience widespread metabolic disturbances, including disruptions in uric acid metabolism (26). Research indicates that more than 50% of hospitalized HF patients exhibit hyperuricemia (27), with elevated blood UA levels closely associated with increased risks of all-cause mortality, cardiovascular death, and composite cardiovascular events (11, 28, 29). However, the specific pathological mechanisms by which UA contributes to the onset and progression of HF are not yet fully understood. Several potential mechanisms have been proposed. Firstly, Xanthine oxidase (XO) is crucial in uric acid metabolism, converting xanthine to uric acid and generating reactive oxygen species (ROS) as byproducts (30). Excessive ROS leads to oxidative stress, activating inflammatory cytokines in vascular endothelial (31) and smooth muscle cells (32), which causes endothelial dysfunction (33) and mechano-energetic uncoupling—an imbalance between myocardial energy metabolism and contraction. This contributes to myocardial hypertrophy, ventricular remodeling, and impaired contractility, worsening heart failure symptoms (34, 35). Secondly, hyperuricemia induces vascular inflammation and promotes the progression of HF through various pathways, including NOD-like receptor heat protein domain-containing protein 3 (36), adenosine monophosphate-activated protein kinase (37), and nuclear factor-kappa B pathways (38). Thirdly, UA can stimulate innate immune responses, further exacerbating the progression of HF (39). These mechanisms underscore the complex role of UA in HF, highlighting the importance of further research to fully elucidate its impact on the disease process.

Albumin is a key antioxidant protein and a marker of nutritional and inflammatory status (40). Low albumin levels are a strong predictor of poor prognosis in HF patients, with persistent inflammation contributing significantly to hypoalbuminemia (12, 41). Our study found that patients with lower albumin levels had higher WBC and neutrophil counts, indicating greater inflammatory activity. Hypoalbuminemia in HF patients can exacerbate circulatory congestion and fluid imbalance, worsening symptoms such as edema. This occurs due to a reduction in intravascular colloid osmotic pressure, increased oxidative stress, heightened inflammation, and an increased susceptibility to infections (42). HF patients often experience malnutrition and inflammatory complications, which lead to increased release of interleukin-2 and interleukin-6. These cytokines not only inhibit hepatic albumin synthesis but also, in conjunction with interferon-alpha, increase vascular endothelial permeability (43). This results in capillary leakage, allowing albumin to escape from the intravascular compartment into the extravascular space, such as the gastrointestinal tract, leading to albumin loss, reduced circulating blood volume, and heart failure decompensation. Furthermore, albumin serves as a crucial carrier protein in the body, facilitating the transport of various drugs. Diuretics, commonly used in the treatment of HF to reduce volume overload, are influenced by albumin through binding interactions (44). With albumin loss, the volume of distribution for diuretics increases, leading to reduced drug concentration and diminished diuretic efficacy, ultimately resulting in fluid retention (45). This phenomenon may also explain the development of diuretic resistance observed in some HF patients.

Combining UA and albumin into a single ratio (UAR) may provide a more nuanced evaluation of oxidative stress, inflammatory responses, and nutritional status, thereby offering a more robust predictor of short-term prognosis in HF patients. Research has demonstrated that elevated levels of UA can stimulate the release of inflammatory cytokines such as interleukin-6 and tumor necrosis factor-alpha (46), which in turn inhibit albumin synthesis, leading to hypoalbuminemia. Additionally, these inflammatory mediators act on vascular endothelial cells, increasing their permeability and causing albumin to leak from the plasma into the interstitial space, further reducing blood albumin levels (47). The interaction between these factors suggests that UAR, which combines the pro-oxidative effects of UA with the antioxidant properties of albumin, may more accurately reflect the overall status of inflammation and oxidative stress in the body. Multiple studies have indicated a strong connection between UAR and the outcomes of cardiovascular diseases like CAD and stroke (15, 19). Higher UAR levels are associated with an increased risk of mortality risk in individuals suffering from ST-segment elevation myocardial infarction (14). Similarly, the study by Çakmak et al. (48) revealed that in patients with non-ST-segment elevation myocardial infarction, higher UAR levels correlate with a higher SYNTAX score, indicating greater severity of CAD. Elevated UAR is also associated with more widespread and severe chronic CAD (15). Furthermore, UAR has been reported as an independent risk factor for the severity and poor short-term prognosis of atherosclerotic stroke. In the present study, we found that when UAR value exceeds 2.11 mg/g, the risk of short-term adverse outcomes in HF patients increases significantly. Our findings are consistent with studies that show combined biomarkers can enhance prognostic accuracy, as UAR appears to capture critical aspects of HF pathophysiology. Therefore, UAR could potentially be used for HF risk stratification, enabling the identification of high-risk individuals for personalized therapeutic interventions in clinical settings.

There are a few limitations in this research. Initially, the retrospective design inherently restricts our capacity to determine a causal relationship between UAR and HF outcomes. Secondly, the study sample was relatively small and derived from a single center, which may limit the generalizability of our findings. To enhance the robustness and applicability of these results, future research should include larger, multicenter studies. Such studies would not only validate these findings but also help to explore the underlying mechanisms driving the observed associations. Future studies should focus on exploring the longitudinal impact of UAR on HF progression and outcomes in diverse patient populations. Additionally, investigating the molecular pathways linking UA and albumin with HF could provide insights into novel therapeutic targets.

## 5 Conclusion

In conclusion, this study underscores the potential of UAR as a novel and valuable prognostic marker in HF patients. Despite the study’s limitations, our findings suggest that incorporating UAR into clinical practice could improve risk prediction and inform more personalized treatment strategies for HF patients.

## Data Availability

The datasets presented in this study can be found in online repositories. The names of the repository/repositories and accession number(s) can be found at: https://physionet.org/content/heart-failure-zigong/1.2/.

## Footnotes

^1^www.mstata.com
