# Supplementary material for: Elevated uric acid to serum albumin ratio: a predictor of short-term outcomes in Chinese heart failure patients
Source: Front Nutr. 2024 Nov 26;11:1481155. doi: 10.3389/fnut.2024.1481155 (PMC11628299; doi:10.3389/fnut.2024.1481155)
Supplement: Supplementary file 1 [file Data_Sheet_1.docx]

Supplementary Material

# Supplementary Figures and Tables

## Supplementary Figures

**
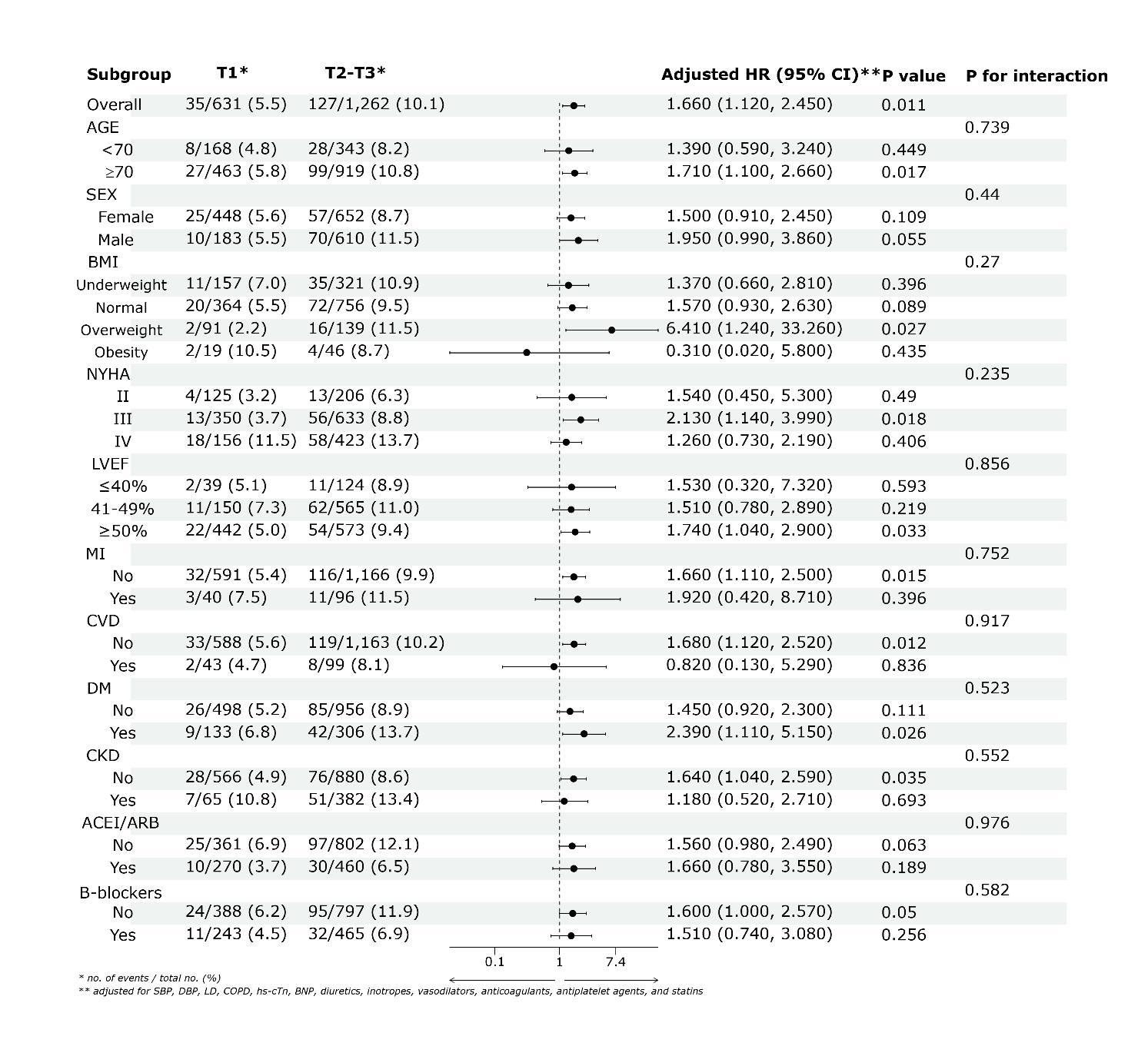
**

**Supplementary Figure 1.** Risk of the 28-day composite endpoint across various subgroups. For all subgroups, the P for interaction > 0.05. Adjusted for SBP, DBP, hs-cTn, BNP, LD, COPD, diuretics, **inotropes**, vasodilators, anticoagulants, antiplatelet agents, and statins. Abbreviations: UAR, uric acid to albumin ratio; ACEI/ARB, angiotensin-converting enzyme inhibitor/angiotensin receptor blocker; BMI, body mass index; NYHA, New York Heart Association; SBP, systolic blood pressure; DBP, diastolic blood pressure; MI, myocardial infarction; CVD, cerebrovascular disease; DM, diabetes mellitus; CKD, chronic kidney disease; COPD, chronic obstructive pulmonary disease; LD, liver disease; hs-cTn, high-sensitivity cardiac troponin; BNP, brain natriuretic peptide; LVEF, left ventricular ejection fraction; T1, Tertile 1; T2, Tertile 2; T3, Tertile 3.

## Supplementary Tables

Supplementary Table 1: Effect of standardized lg(UAR) level on survival: adjusted hazard ratios from segmented cox regression analysis

| **Characteristic** | **HR per SD** | **95% CI** | ***p*-value** |
| --- | --- | --- | --- |
| lg(UAR) (< 0.324) | 1.24 | 0.93, 1.65 | 0.15 |
| lg(UAR) (≥ 0.324) | 1.40 | 1.21, 1.62 | <0.001 |
| HRs were adjusted for diuretics, **inotropes**, vasodilators, ACEI/ARB, B-blockers, anticoagulants, antiplatelet agents, statins, AGE, SEX, BMI, NYHA, SBP, DBP, MI, CVD, DM, CKD, LD, COPD, hs-cTn, and BNP.  Abbreviations: HR, hazard ratio; CI, confidence interval; BMI, body mass index; NYHA, New York Heart Association; SBP, systolic blood pressure; DBP, diastolic blood pressure; MI, myocardial infarction; CVD, cerebrovascular disease; DM, diabetes mellitus; CKD, chronic kidney disease; COPD, chronic obstructive pulmonary disease; LD, liver disease; hs-cTn, high-sensitivity cardiac troponin; BNP, brain natriuretic peptide; UAR, uric acid to albumin ratio; ACEI/ARB, angiotensin-converting enzyme inhibitor/angiotensin receptor blocker; Β-blocker, β-receptor blockers. | | | |

Supplementary Table 2: Association between lg(UAR) and clinical outcomes in heart failure patients with LVEF data

| **Variables** | **Model1** | |  | **Model2** | |  | **Model3** | |  | **Model4** | |  | **Model5** | |
| --- | --- | --- | --- | --- | --- | --- | --- | --- | --- | --- | --- | --- | --- | --- |
|  | **HR (95%CI)** | ***P*** |  | **HR (95%CI)** | ***P*** |  | **HR (95%CI)** | ***P*** |  | **HR (95%CI)** | ***P*** |  | **HR (95%CI)** | ***P*** |
| Lg(UAR) | 1.513 (1.299, 1.762) | <0.001 |  | 1.495 (1.279, 1.747) | <0.001 |  | 1.325 (1.120, 1.569) | 0.001 |  | 1.280 (1.085, 1.509) | 0.003 |  | 1.266 (1.065, 1.505) | 0.008 |
| Lg(UAR) Tertiles | |  |  |  |  |  |  |  |  |  |  |  |  |  |
| Tertile1 | 1.000 (Reference) |  |  | 1.000 (Reference) |  |  | 1.000 (Reference) |  |  | 1.000 (Reference) |  |  | 1.000 (Reference) |  |
| Tertile2 | 1.304 (0.838, 2.028) | 0.239 |  | 1.269 (0.814, 1.978) | 0.294 |  | 1.193 (0.760, 1.872) | 0.443 |  | 1.130 (0.722, 1.768) | 0.593 |  | 1.113 (0.709, 1.748) | 0.641 |
| Tertile3 | 2.433 (1.638, 3.615) | <0.001 |  | 2.332 (1.559, 3.489) | <0.001 |  | 1.869 (1.215, 2.876) | 0.004 |  | 1.674 (1.095, 2.557) | 0.017 |  | 1.620 (1.043, 2.518) | 0.032 |
| *P* for trend |  | <0.001 |  |  | <0.001 |  |  | 0.002 |  |  | 0.007 |  |  | 0.018 |
| Model1: Crude | | | | | | | | | | | | | | |
| Model2: Adjust: AGE, SEX, BMI | | | | | | | | | | | | | | |
| Model3: Adjust: AGE, SEX, BMI, NYHA, SBP, DBP, MI, CVD, DM, CKD, LD, COPD, LVEF | | | | | | | | | | | | | | |
| Model4: Adjust: diuretics, **inotropes**, vasodilators, ACEI/ARB, B-blockers, anticoagulants, antiplatelet agents, statins, AGE, SEX, BMI, NYHA, SBP, DBP, MI, CVD, DM, CKD, LD, COPD, LVEF | | | | | | | | | | | | | | |
| Model5: Adjust: diuretics, **inotropes**, vasodilators, ACEI/ARB, B - blockers, anticoagulants, antiplatelet agents, statins, AGE, SEX, BMI, NYHA, SBP, DBP, MI, CVD, DM, CKD, LD, COPD, hs-cTn, BNP, LVEF | | | | | | | | | | | | | | |
| Abbreviations: HR: hazard ratio; CI: confidence interval; BMI, body mass index; NYHA, New York Heart Association; SBP, systolic blood pressure; DBP, diastolic blood pressure; MI, myocardial infarction; CVD, cerebrovascular disease; DM, diabetes mellitus; CKD, chronic kidney disease; COPD, chronic obstructive pulmonary disease; LD, liver disease; hs-cTn, high-sensitivity cardiac troponin; BNP, brain natriuretic peptide; UAR, uric acid to albumin ratio; ACEI/ARB, angiotensin-converting enzyme inhibitor/angiotensin receptor blocker; Β-blocker, β-receptor blockers; LVEF, left ventricular ejection fraction. | | | | | | | | | | | | | | |
